# Cyclic helix B peptide ameliorates acute myocardial infarction in mice by inhibiting apoptosis and inflammatory responses

**DOI:** 10.1038/s41420-019-0161-y

**Published:** 2019-03-18

**Authors:** Cheng Yang, Chao Zhang, Jianguo Jia, Lingyan Wang, Weitao Zhang, Jiawei Li, Ming Xu, Ruiming Rong, Tongyu Zhu

**Affiliations:** 1 Department of Urology, Zhongshan Hospital, Fudan University, Shanghai, 200032 China; 2 Shanghai Key Laboratory of Organ Transplantation, Shanghai, 200032 China; 3 Zhangjiang Institute of Fudan University, Shanghai, 201203 China; 4 Department of Cardiology, Zhongshan Hospital, Fudan University, Shanghai, 200032 China; 5 Shanghai Institute of Cardiovascular Diseases, Shanghai, 200032 China; 6 Biomedical Research Center, Zhongshan Hospital, Fudan University, Shanghai, 200032 China; 7 Department of Transfusion, Zhongshan Hospital, Fudan University, Shanghai, 200032 China

## Abstract

Cyclic helix B peptide (CHBP) is a peptide derivant of erythropoietin with powerful tissue-protective efficacies in a variety of organ injuries, but without erythropoietic effect. However, the role of CHBP in acute myocardial infarction (AMI) and related mechanisms are not studied yet. In this study, we found in a murine AMI model that the administration of CHBP could ameliorate cardiac injury, increase the survival rate, inhibit cardiomyocyte apoptosis, improve cardiac function and remodeling, and reduce the expression of inflammatory cytokines in the serum and kidney tissue both at 24 h and 8 weeks following AMI. This study suggests that CHBP has the potential to be used as an effective drug in the treatment of AMI.

## Introduction

It is reported by World Health Organization that 17 million deaths (in 57 million global deaths) are attributed to cardiovascular diseases every year, among which ischemic heart disease and congestive heart failure remain the two leading causes^1^. Heart failure results from the reduction or even blockade of blood supply and apoptosis of cardiomyocytes in infarcted areas following acute myocardial infarction (AMI). Given that the terminally differentiated cardiomyocytes are not able to regenerate, the remaining viable cardiomyocytes in non-infarcted areas have to be forced to share a heavier burden to maintain a sufficient cardiac output, thereby leading to myocardial remodeling. In severe cases, however, the remodeling decompensates and becomes pathogenic, and ultimately leads to heart failure^2^. Thus, novel therapeutic approaches to ameliorating the development of AMI are critical for the improvement of prognosis in patients.

Since the early 1990s, it has emerged that endogenous erythropoietin (EPO) has cytoprotective effects in a wide variety of tissues, including brain, kidney, and heart^3^. Numerous experimental evidences showed cardioprotective effects of EPO in animal models of AMI. However, these findings are not supported by recent clinical trials designed to investigate the safety and efficacy of EPO in the patients with AMI^4^. To avoid the erythropoietic side effect of EPO, Brines et al.^5^ firstly reported a nonerythropoietic helix B surface peptide (HBSP) in 2008. In the next period, we and others demonstrated the tissue-protective function of HBSP in a variety of organs, such as kidney^6–8^, heart^9,10^, and brain^11^. However, HBSP demonstrated short half-life in human plasma and liver, and fast degradation in vivo. The instability of HBSP restricts its application in vivo^5,12^. Recently, based on the amino acid sequence of HBSP, we designed and synthesized a novel thioether-cyclized helix B peptide (CHBP) with the increased resistance to proteolytic degradation, improved tissue-protective potency, and decreased administrative frequency and dosage^12,13^. Our previous studies demonstrated that CHBP could protect against ischemia-reperfusion induced kidney injury and carbon tetrachloride induced liver injury^14–17^. However, whether CHBP could also function in ameliorating cardiac injury following AMI has not been investigated yet.

Therefore, we designed the present study to determine the role of CHBP in AMI therapy and reveal its cardioprotective mechanisms. We also aim to investigate the changes of morphology and cardiac function, as well as the regulation of apoptosis and inflammation by CHBP treatment.

## Results

### CHBP reduced tissue injury, myocardial infarct size, and the expression of CK-MB in the serum

**Fig. 1:**
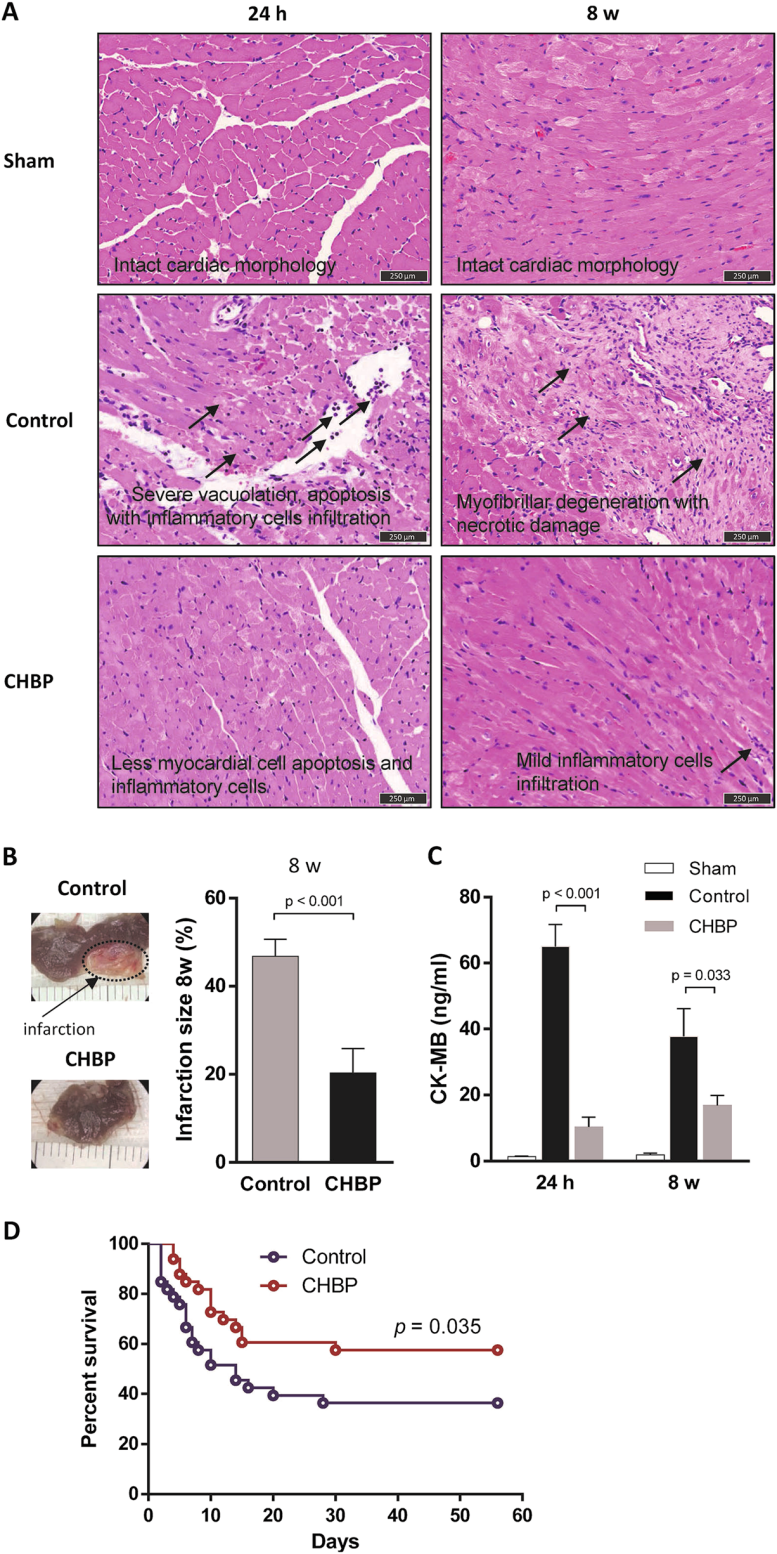
CHBP ameliorated cardiac injury. The H&E staining showed CHBP significantly attenuated cardiac injury in terms of less vacuolation, myofibrillar degeneration, apoptosis and necrosis, as well as less inflammatory cells infiltration post 24 h and 8-weeks AMI (**a**). Ischemia induced significant myocardial injury as denoted by the infarct size in control mice. In contrast, infarct size was significantly reduced in CHBP-treated mice, when compared with control mice (**b**). The expression of CK-MB in the serum was also significantly decreased by CHBP treatment (**c**). In addition, CHBP also remarkably increased the survival rate after AMI (**d**)

The H&E-stained myocardial sections showed little apoptotic cells and infiltrated inflammatory cells in CHBP-treated mice following 24 h ischemia injury. However, severe vacuolation and apoptosis with inflammatory cell infiltration were noted in the control group. Following 8-weeks ischemia injury, myofibrillar degeneration with necrotic damage was observed in the control group, and CHBP ameliorated tissue injury with mild inflammatory cell infiltration (Fig. 1a). We also examined the role of CHBP in the reduction of myocardial infarct size following 8-weeks ischemia injury. As shown in Fig. 1b, ischemia induced a significant myocardial injury as denoted by the infarct size in control mice. In contrast, the infarct size was significantly reduced in CHBP-treated mice, when compared with control mice (Fig. 1b). We, furthermore, examined the level of CK-MB in the serum, and the result showed that CHBP significantly decreased the CK-MB level compared to the control group (Fig. 1c). Survival analysis demonstrated that CHBP significantly increased the survival rate after AMI (Fig. 1d).

### CHBP-attenuated cardiomyocyte apoptosis

**Fig. 2:**
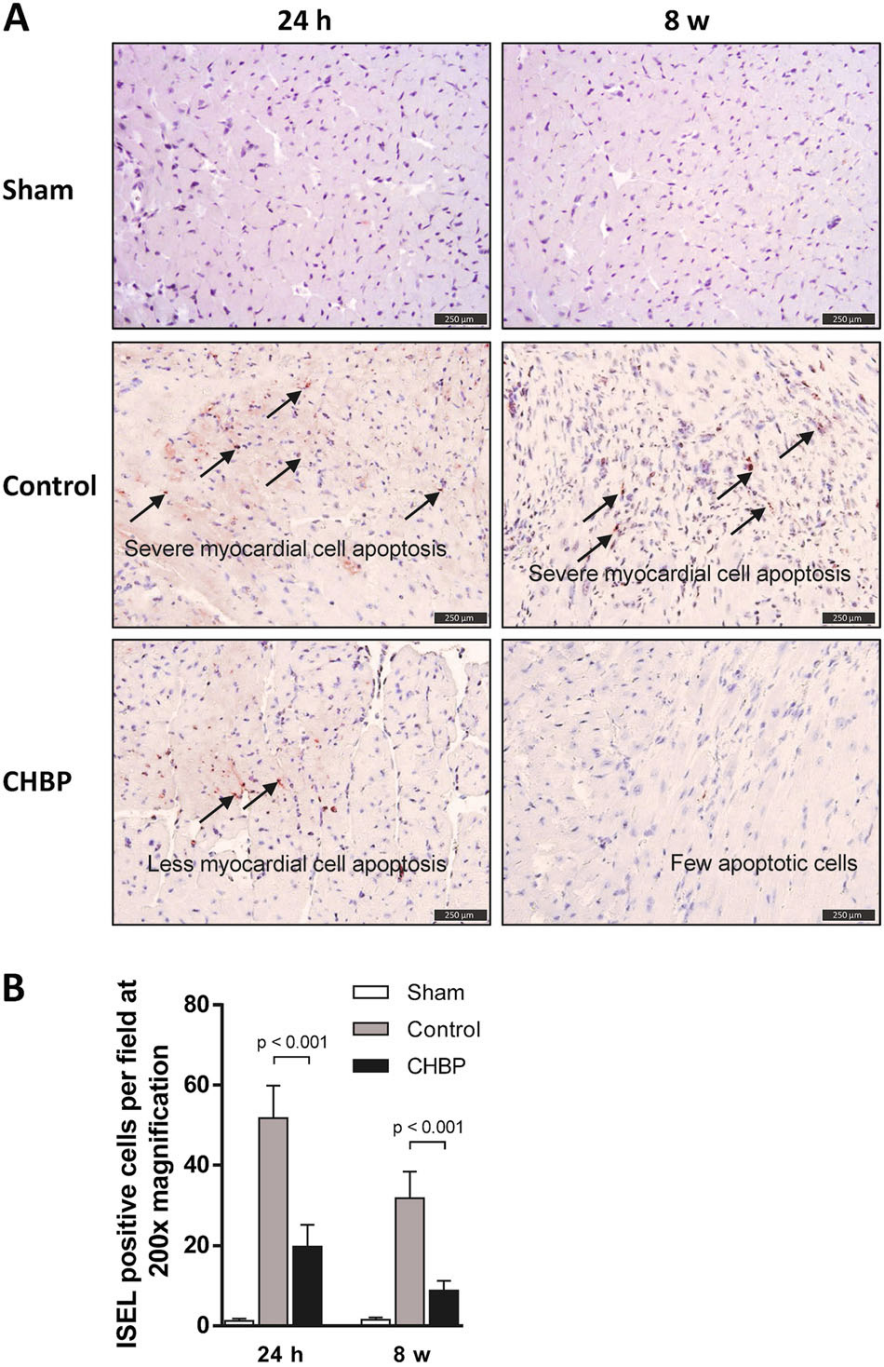
CHBP-attenuated cardiaomyocyte apoptosis. Apoptotic cells were labeled by TUNEL assay (**a**). Ischemia significantly increased the ISEL-positive apoptotic cells in myocardium in the control group compared with that in the sham group. However, myocardial apoptotic cells were markedly reduced by CHBP compared with the control mice (**b**). TUNEL: terminal-deoxynucleoitidyl transferase mediated nick end-labeling; ISEL: in situ end-labeling

Cardiomyocyte apoptosis contributes to myocardial ischemia injury^18^. We examined whether CHBP would attenuate myocardial apoptosis following short-term and long-term ischemia injury. Figure 2a showed that ischemia significantly increased the ISEL-positive apoptotic cells in myocardium in the control group compared with the sham group. However, myocardial apoptotic cells were markedly reduced by CHBP compared with the control mice (Fig. 2b).

### CHBP improved left ventricular function and remodeling

**Fig. 3:**
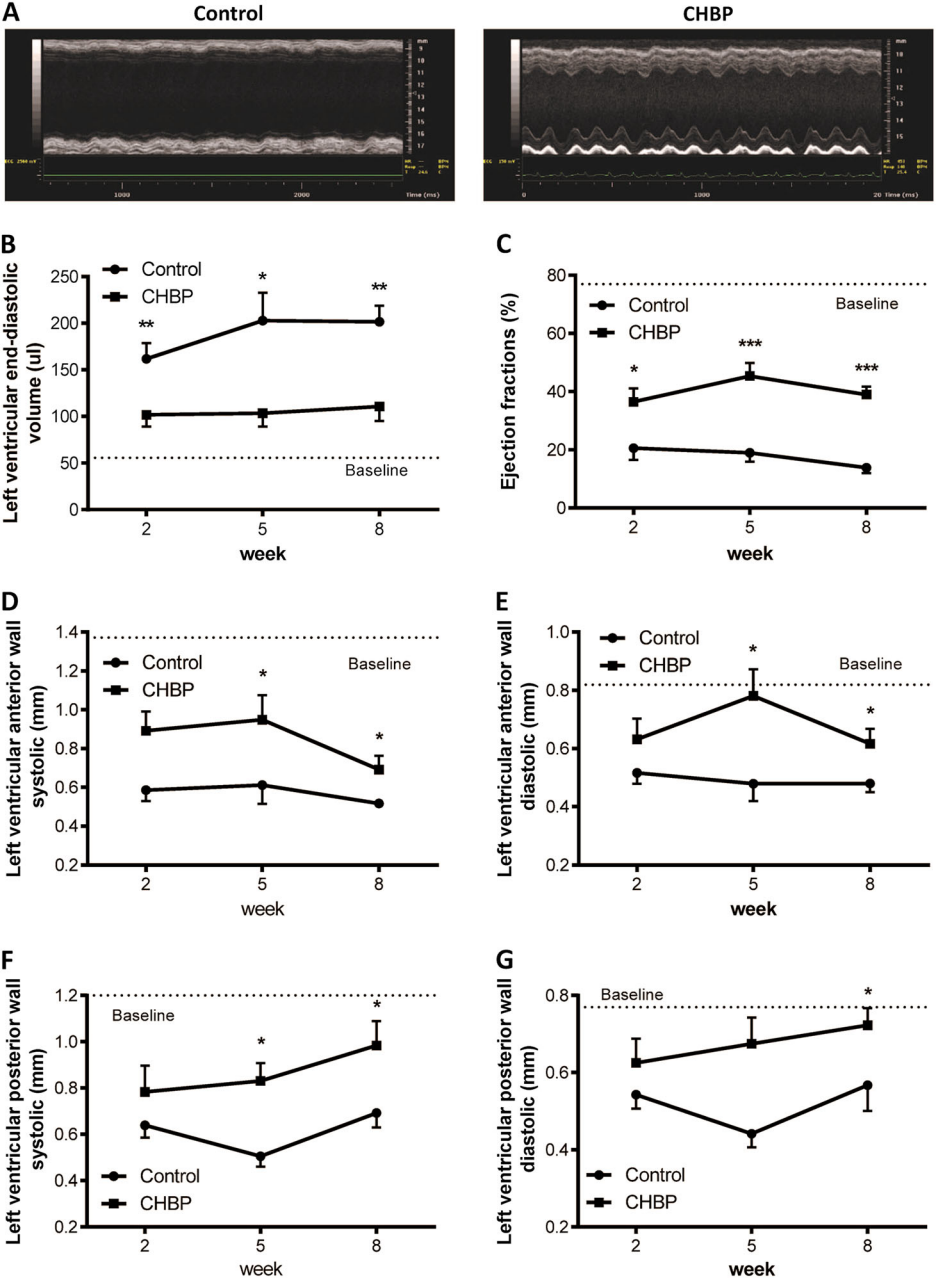
CHBP improved left ventricular function and remodeling. Representative M-mode images and echocardiographic parameters in control and CHBP-treated mice after 8-weeks ischemia injury are shown (**a**). The left ventricular end-diastolic volume was significantly larger in the control group compared to that in the CHBP group (**b**). The ejection fraction was also remarkably improved in the CHBP group (**c**). The systolic (**d**) and diastolic (**e**) left ventricular anterior wall were significantly thicker by CHBP treatment, as well as the systolic (**f**) and diastolic (**g**) posterior wall thickness. The dot line represents baseline parameters, which were measured by sham mice

Representative M-mode images and echocardiographic parameters in control and CHBP-treated mice after 8-weeks ischemia injury were shown in Fig. 3a. The left ventricular end-diastolic volume was significantly larger in the control group than in the CHBP group (Fig. 3b). The ejection fraction was also remarkably increased in the CHBP group (Fig. 3c). The systolic (Fig. 3d) and diastolic (Fig. 3e) left ventricular anterior wall were significantly thicker following CHBP treatment, as well as the systolic (Fig. 3f) and diastolic (Fig. 3g) posterior wall thickness.

### CHBP modulated the inflammatory responses and chemokines expression

**Fig. 4:**
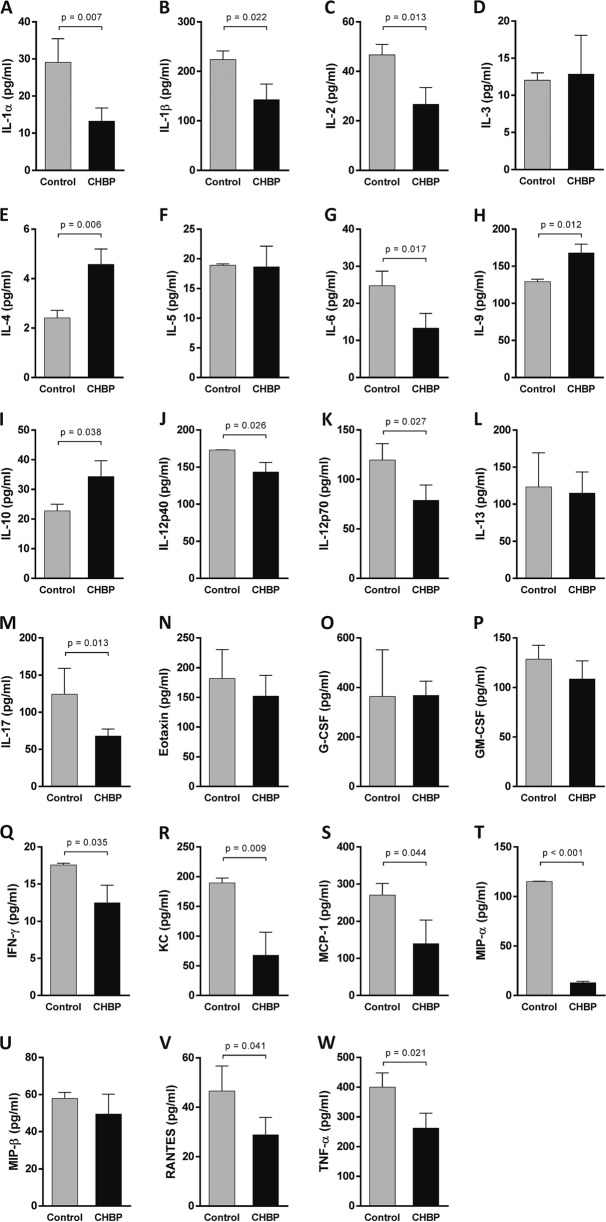
The inflammatory cytokines and chemokines expression at 24 h post AMI. Pro-inflammatory cytokines, including IL-1α (**a**), IL-1β (**b**), IL-2 (**c**), IL-6 (**g**), IL-12p40 (**j**), IL-12p70 (**k**), IL-17 (**m**), IFN-γ (**q**), MCP-1 (**s**), MIP-α (**t**), and TNF-α (**w**) were significantly decreased by CHBP treatment. In contrast, the anti-inflammatory cytokines IL-4 (**e**), IL-9 (**h**), and IL-10 (**i**) were significantly increased in the CHBP-treated group compared to those in the control group. The chemokines KC/CXCL1 (**r**) and RANTES/CCL5 (**v**) were significantly increased by CHBP. The expressions of IL-3 (**d**), IL-5 (**f**), IL-13 (**l**), Eotaxin (**n**), G-CSF (**o**), GM-CSF (**p**) and MIP-β (**u**) were not significantly different between the control and CHBP group

**Fig. 5:**
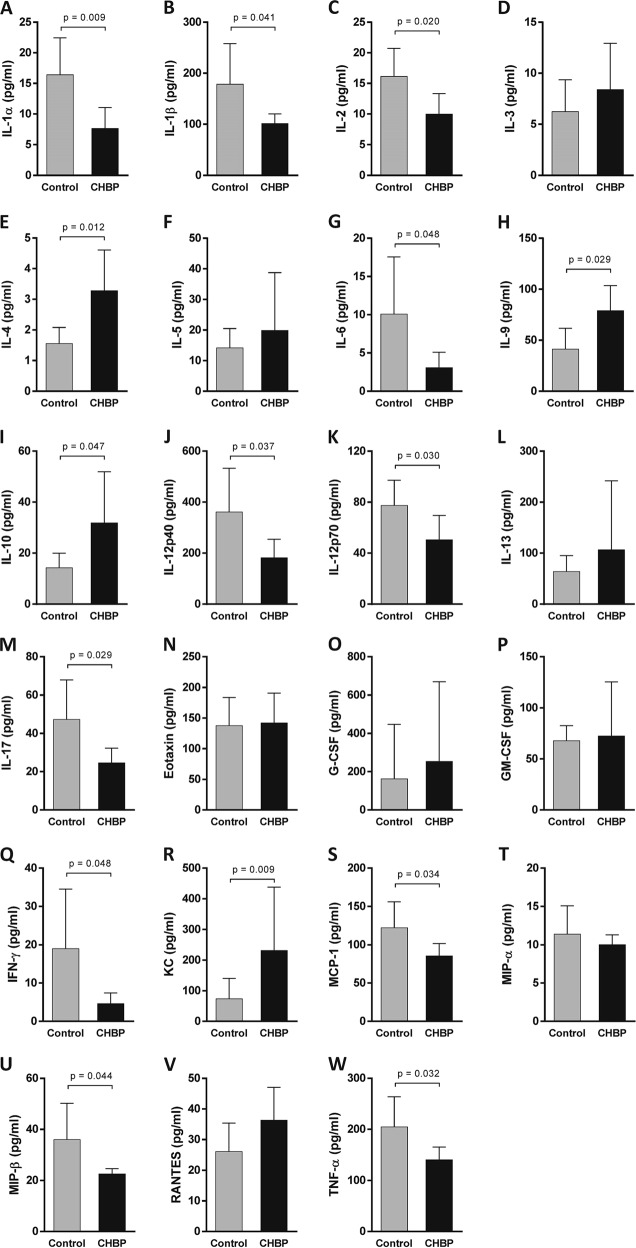
The inflammatory cytokines and chemokines expression at 8-weeks post AMI. The expression of IL-1α (**a**), IL-1β (**b**), IL-2 (**c**), IL-6 (**g**), IL-12p40 (**j**), IL-12p70 (**k**), IL-17 (**m**), IFN-γ (**q**), MCP-1 (**s**), MIP-β (**u**), and TNF-α (**w**) were significantly decreased by CHBP treatment, whereas IL-4 (**e**), IL-9 (**h**), and IL-10 (**i**) were remarkably by CHBP treatment. The KC/CXCL1 (**r**) expression was significantly increased in the CHBP group. The expressions of IL-3 (**d**), IL-5 (**f**), IL-13 (**l**), Eotaxin (**n**), G-CSF (**o**), GM-CSF (**p**), MIP-α (**t**) and RANTES (**v**) were not significantly different between the control and CHBP group

To evaluate the systemic inflammatory response, we detected pro- and anti-inflammatory cytokines in peripheral blood using Luminex assay. At 24 h post AMI, pro-inflammatory cytokines, including interlukin (IL)-1α (Fig. 4a), IL-1β (Fig. 4b), IL-2 (Fig. 4c), IL-6 (Fig. 4g), IL-12p40 (Fig. 4j), IL-12p70 (Fig. 4k), IL-17 (Fig. 4m), interferon  (IFN)-γ (Fig. 4q), monocyte chemoattractant protein-1 (MCP-1) (Fig. 4s), macrophage inflammatory protein-1α (MIP-α) (Fig. 4t), and tumor necrosis factor (TNF)-α (Fig. 4w) were significantly decreased by CHBP treatment. In contrast, the anti-inflammatory cytokines IL-4 (Fig. 4e), IL-9 (Fig. 4h), and IL-10 (Fig. 4i) were significantly increased in the CHBP-treated group compared to those in the control group. The chemokines KC/CXCL1 (Fig. 4r) and RANTES/CCL5 (Fig. 4v) were significantly increased by CHBP. Eight weeks post AMI, the change of inflammatory cytokines was similar to that at 24 h. IL-1α (Fig. 5a), IL-1β (Fig. 5b), IL-2 (Fig. 5c), IL-6 (Fig. 5g), IL-12p40 (Fig. 5j), IL-12p70 (Fig. 5k), IL-17 (Fig. 5m), IFN-γ (Fig. 5q), MCP-1 (Fig. 5s), MIP-β (Fig. 5u), and TNF-α (Fig. 5w) were significantly decreased by CHBP treatment, whereas IL-4 (Fig. 5e), IL-9 (Fig. 5h), and IL-10 (Fig. 5i) were remarkably by CHBP treatment. Unlike to the 24 h post AMI, the KC/CXCL1 expression was significantly increased in the CHBP group (Fig. 5r).

**Fig. 6:**
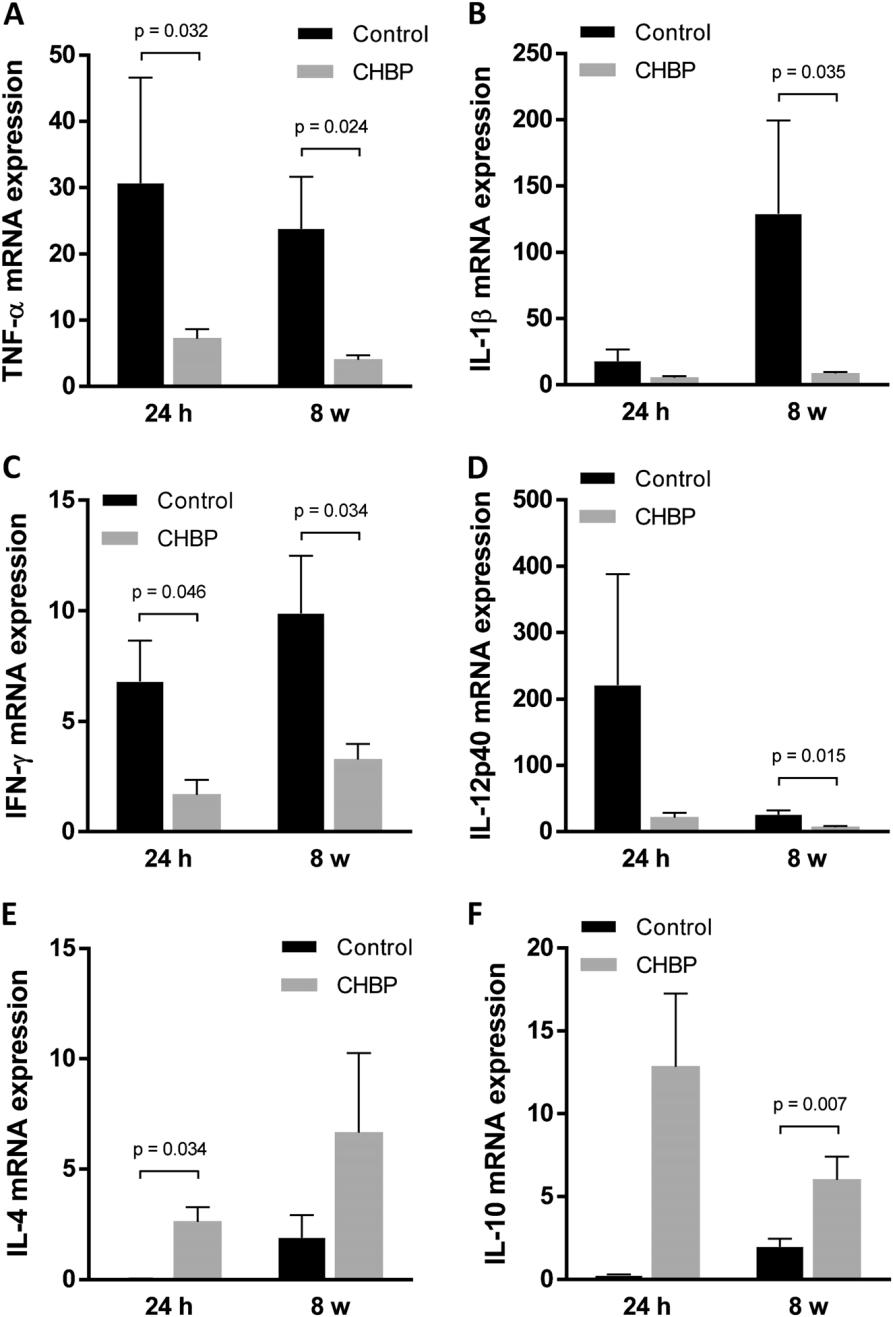
The inflammatory cytokines expression at mRNA level post AMI. The mRNA expression of TNF-α (**a**) and IFN-γ (**c**) was significantly decreased by CHBP treatment at both 24 h and 8 weeks. IL-1β (**b**) and IL-12p40 (**d**) mRNA level was significantly reduced in myocardium. The mRNA expression of IL-4 (**e**) and IL-10 (**f**) was significantly increased at 24 h and 8 weeks in the CHBP-treated group, respectively

To evaluate the local inflammation responses, we also examined the typical inflammatory cytokines mRNA expression in the myocardium by using RT-qPCR. The mRNA expression of TNF-α (Fig. 6a) and IFN-γ (Fig. 6c) was significantly decreased by CHBP treatment at both 24 h and 8 weeks. IL-1β (Fig. 6b) and IL-12p40 (Fig. 6d) mRNA level was significantly reduced in the myocardium. The mRNA expression of IL-4 (Fig. 6e) and IL-10 (Fig. 6f) was significantly increased at 24 h and 8 weeks in the CHBP-treated group, respectively.

## Discussion

This study demonstrated that the novel peptide CHBP exerted cardioprotective effect in a murine AMI model. CHBP significantly ameliorated histological injury and left ventricular remodeling. CHBP also inhibited cardiomyocytes apoptosis and inflammation.

CHBP is a thioether-cyclized peptide with conformational constraining and firstly reported by our group in a kidney ischemia-reperfusion model^12^. Although its parent protein erythropoietin (EPO) has been demonstrated to alleviate heart injury and promote heart repair in the animal studies about AMI^19,20^, the large REVEAL study showed no reduction of infarct size following EPO treatment^21^. Recently, a meta-analysis indicated that short-term administration of EPO in the patients with myocardial infarction did not result in an improved cardiac function, reduced infarct size and lower all-cause mortality^22^. Similarly, the results of a randomized clinical trial indicated that low-dose EPO also did not improve cardiac function in the patients with ST-segment elevation myocardial infarction^23^. Low-dose EPO therapy may not be the choice of treatment for the patients with AMI, while a higher dose of EPO might be more effective^24^. However, high-dose EPO therapy would take higher risks of side effects, such as erythrocytosis, thrombogenesis and hypertension^25,26^. To avoid the abovementioned side effects while maintain the tissue-protective efficacy of EPO, Brines et al. firstly reported a linear peptide helix B surface peptide (HBSP), which entirely replicated the cardioprotective properties from the parent erythropoietin, but without erythropoiesis^9,10^. Unfortunately, HBSP was proved to be very unstable both in vivo and in vitro. Owing to its short plasma half-life (about 9 min), linear HBSP needs to be frequently administered with high doses to achieve tissue-protective effects, and that would restrict its clinical application^12^. Therefore, we designed and synthesized CHBP with better stability and stronger tissue-protective property^16,27–30^. This study confirmed the cardioprotective effect of CHBP.

There are some limitations in the present study. For instance, the molecular mechanism involved in the regulation of apoptosis and inflammation by CHBP needs to be further investigated. To evaluate the long-term cardioprotective effects of CHBP from bench to bedside, more samples and longer observation are still required.

In conclusion, our study reveals that the novel peptide CHBP protects heart against AMI in terms of better heart function, less cardiomyocytes injury and apoptosis, and low level of inflammation.

## Materials and methods

### Animals and surgery

Male C57B6 mice (weighing 20–25 g) were obtained from Shanghai Slac Lab Animal, Co., Ltd., and bred in an experimental animal room of SPF grade. Mice were anaesthetized (2% isoflurane), intubated and ventilated, and then were placed on an adjustable heating pad to maintain a core temperature of 36–37 °C. The heart was exposed through the fourth intercostal space separated with an adjustable microretractor (Medicon eG, Tuttlingen, Germany) and the left anterior descendent coronary artery was (LAD) ligated using 10/0 silk suture (Ethicon Endo-surgery, OH, USA), 1 mm distal to left atrial appendage. Successful performance of coronary occlusion was verified by the visible pale of afflicted tissue and QRS alterations within the first seconds of occlusion. The sham-operated animals underwent the same surgical procedures except that the suture passing the LAD was not fastened. After surgery, buprenorphine (0.05 mg/kg per 6 h, subcutaneously) was given for 48 h. Immediately after surgery, the mice were injected with 30 μg/kg CHBP i.p. for a single dose. For short-term therapeutic effect evaluation, mice were randomly divided into three groups (*n* = 10): (1) sham group; (2) control group; (3) CHBP group: with one dose of CHBP administration. All mice were sacrificed at 24 h post surgery. For long-term therapeutic effect evaluation, mice were also randomly divided into three groups (*n* = 33): (1) sham group; (2) control group; (3) CHBP group: with 30 μg/kg CHBP administration three times a week. All mice were sacrificed at 8-weeks post surgery. All animal procedures were performed according to the guidelines of the Care and Use of the Laboratory Animal Ethical Commission of Fudan University.

### Histology

Hearts were arrested with 15% KCl and sectioned into 3–4 transverse slices, cut parallel to the atrioventricular ring. Each slice was fixed with 4% (v/v) buffered formalin, embedded in paraffin, and sectioned into 5 μm sections. Serial sections were stained with hematoxylin and eosin (H&E; Sigma).

### Enzyme-linked immunosorbent assay (ELISA) assay

Creatine kinase-MB **(**CK-MB) was assayed in duplicate using sandwich ELISA (Quantikine Kit for Mice CK-MB Immunoassay; Uscn Life Science Inc., Houston, TX, USA). The sample preparation and procedure were performed according to the manufacturer’s instructions.

### In situ end-labeling (ISEL) apoptotic cells

ISEL apoptotic cells were detected using a TUNEL Apoptosis Detection Kit (Millipore, MA, USA). Paraffin sections of 4 µm were digested by 40 µg/mL of proteinase K (EMD Chemicals, NJ, USA) for 15 min at 37 °C, incubated with TdT and digoxigenin-dUTP at 37 °C for 60 min, and transferred to a wash/stop buffer for 30 min. After adding anti-digoxigenin-peroxidase complex for 30 min, the tissue sections immersed in buffer were developed by 3′-amino-9-ethylcarbazole (AEC, DAKO, Carpinteria, USA) substrate (dark red color). Apoptotic cells were examined at  × 400 magnification in 20 fields for semi-quantitation.

### Transthoracic echocardiographic analysis

Echocardiographic measurements were performed with a Vevo 770 system (Visual Sonics, Toronto, Ontario, Canada) applied to the shaved chest wall of mice anesthetized with isoflurane. All measurements, averaged for three consecutive cardiac cycles, were performed by an experienced technician and reviewed by a cardiologist; all were blinded to the treatment groups. The ejection fraction (EF), left ventricular end-diastolic volume, left ventricular systolic and diastolic anterior, and posterior wall thickness were measured in M-mode recordings.

### Cytokine multiplex analysis

Serum samples were analyzed for 23 cytokines using a Bio-Plex Multiplex System (Bio-Rad, Hercules, CA, USA) according to the manufacturer’s protocol.

### Real-time quantitative polymerase chain reaction (RT-qPCR)

Total RNA was extracted from mice kidneys with Trizol reagent (Invitrogen, Carlsbad, USA) according to the manufacturer’s instructions. Total RNA (3 to 5 µg) was transcribed into complementary DNA by the Superscript II reverse transcriptase (Invitrogen) and random primer oligonucleotides (Invitrogen). Gene-specific primers for mice, such as TNF-α, IL-1β, IFN-γ, IL-12p40, IL-4, and IL-10 and GAPDH, were designed based on the sequences available from PubMed (Table S1 contains the list of sequences). RT-qPCR was performed in a MasterCycler RealPlex4 system (Eppendorf, Hamburg, Germany) in combination with the Absolute QPCR SYBR Green premix (Takara Bio Inc., Tokyo, Japan). After a hot start (30 s at 95 °C), the amplification parameters were as follows: 5 s at 95 °C, 30 s at 55 °C, and 60 s at 72 °C for 45 cycles. The expression levels, which were normalized with GAPDH, were calculated according to the housekeeping gene GAPDH using the 2^−ΔΔCt^ method.

### Statistical analysis

Data are presented as mean ± standard deviation (SD). Statistical analysis (SPSS 18.0 software, SPSS Inc, Armonk, NY, USA) was performed with the two-tailed independent Student’s *t*-test after the demonstration of homogeneity of variance with the *F*-test or one-way ANOVA for more than two groups. The Scheffe test was used for post-hoc analysis. Statistical significance was set as *p* < 0.05.
